# Mitochondria‐associated membranes (MAMs): molecular organization, cellular functions, and their role in health and disease

**DOI:** 10.1002/2211-5463.70121

**Published:** 2025-10-10

**Authors:** Viet Bui, Maryline Santerre, Natalia Shcherbik, Bassel E Sawaya

**Affiliations:** ^1^ FELS Cancer Institute for Personalized Medicine, Lewis Katz School of Medicine Temple University Philadelphia PA USA; ^2^ Department for Cell and Molecular Biology Rowan‐Virtua School of Osteopathic Medicine Stratford NJ USA; ^3^ Department of Cancer and Cellular Biology, Lewis Katz School of Medicine Temple University Philadelphia PA USA; ^4^ Department of Neural Cells, Lewis Katz School of Medicine Temple University Philadelphia PA USA

**Keywords:** calcium signaling, cellular stress responses, ER–mitochondria contact sites, lipid metabolism, mitochondria‐associated membranes, neurodegeneration

## Abstract

Mitochondria‐associated membranes (MAMs) are specialized contact sites between the endoplasmic reticulum (ER) and mitochondria that maintain cellular homeostasis through precisely orchestrated molecular mechanisms. These dynamic interfaces are maintained at 10–50 nm distances by complex tethering proteins, including the core IP3R–GRP7 5–VDAC1 complex and regulatory proteins, such as the sigma‐1 receptor. MAMs coordinate multiple essential cellular processes: lipid synthesis and transfer, calcium signaling, metabolic regulation, and quality control through autophagy and mitophagy. Recent advances in super‐resolution microscopy and proteomics have revealed that MAM dysfunction drives pathogenesis across various diseases. In Alzheimer's disease, disrupted MAM spacing directly affects Aβ production and mitochondrial function, while in Parkinson's disease, α‐synuclein accumulation at MAMs impairs phosphatidylserine metabolism and mitochondrial dynamics. Beyond neurodegeneration, MAMs play crucial roles in metabolic disorders, cancer progression, and viral infections. This review provides mechanistic insights into MAM biology, from molecular organization to disease pathogenesis, integrating structural analyses with dynamic visualization approaches. We examine emerging therapeutic strategies targeting MAM‐associated pathways and highlight their potential in treating complex diseases.

## Graphical abstract

Mitochondria‐associated membranes (MAMs) are contact sites between the endoplasmic reticulum and mitochondria that regulate calcium signaling, lipid metabolism, autophagy, and stress responses. This review outlines their molecular organization, roles in cellular homeostasis, and how dysfunction drives neurodegeneration, metabolic disease, cancer, and aging.

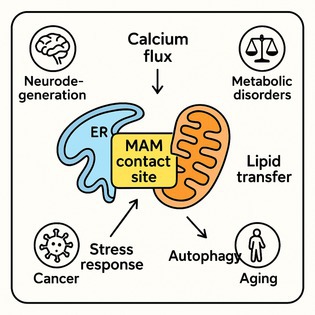

**Abbreviations**
AD: Alzheimer's disease
ALS: amyotrophic lateral sclerosis
AMPK: AMP‐activated protein kinase
APP: amyloid precursor protein
ATP: adenosine triphosphate
Ca^2+^
: calcium ion
cART: combination antiretroviral therapy
DRP1: dynamin‐related protein 1
ER stress: endoplasmic reticulum stress
ER: endoplasmic reticulum
ER: –mitochondria contacts, endoplasmic reticulum–mitochondria contact sites
ERK: extracellular signal‐regulated kinase
GSK3β: glycogen synthase kinase 3 beta
IP3R: inositol 1,4, 5‐trisphosphate receptor
IRE1: inositol‐requiring enzyme 1
ISR: integrated stress response
MAMs: mitochondria‐associated membranes
mPTP: mitochondrial permeability transition pore
mtDNA: mitochondrial DNA
OPA1: optic atrophy 1
PD: Parkinson's disease
PERK: protein kinase RNA‐like ER kinase
PKM2: pyruvate kinase M2
PTPIP51: protein tyrosine phosphatase‐interacting protein 51
ROS: reactive oxygen species
SERCA: sarco/endoplasmic reticulum Ca^2+^‐ATPase
UPR: unfolded protein response
VAPB: vesicle‐associated membrane protein‐associated protein B
VDAC: voltage‐dependent anion channel

Mitochondria‐associated membranes (MAMs) are specialized contact sites that facilitate critical communication between the endoplasmic reticulum (ER) and mitochondria [1]. First observed by Jean Vance in 1990 via electron microscopy [2], these domains were later biochemically defined as hubs for phospholipid synthesis and transfer. Advances in super‐resolution microscopy and proximity labeling have since revealed MAMs as dynamic platforms regulating lipid metabolism, calcium signaling, autophagy, and cellular stress responses [3].

The molecular architecture of MAMs relies on the tight spatial organization of the ER and mitochondria, maintaining an intermembrane distance of 10–50 nm [4, 5]. This precise alignment enables molecular exchange without fusion and is regulated by a tethering complex consisting of:

- Inositol 1,4,5‐trisphosphate receptor (IP3R) on the ER membrane,
- Glucose‐regulated protein 75 (GRP75) as a molecular linker, and
- Voltage‐dependent anion channel 1 (VDAC1) on the outer mitochondrial membrane [6].

Additional components stabilize these contacts and contribute to functional specificity. ER‐resident proteins include Mitofusin 2 (MFN2), vesicle‐associated membrane protein‐associated protein B (VAPB), B‐cell receptor‐associated protein 31 (BAP31), and phosphofurin acidic cluster sorting protein 2 (PACS2). Mitochondrial components include protein tyrosine phosphatase‐interacting protein 51 (PTPIP51), Mitofusin 1/2 (MFN1/2), and mitochondrial fission 1 protein (FIS1) (Fig. 1).

**Fig. 1:**
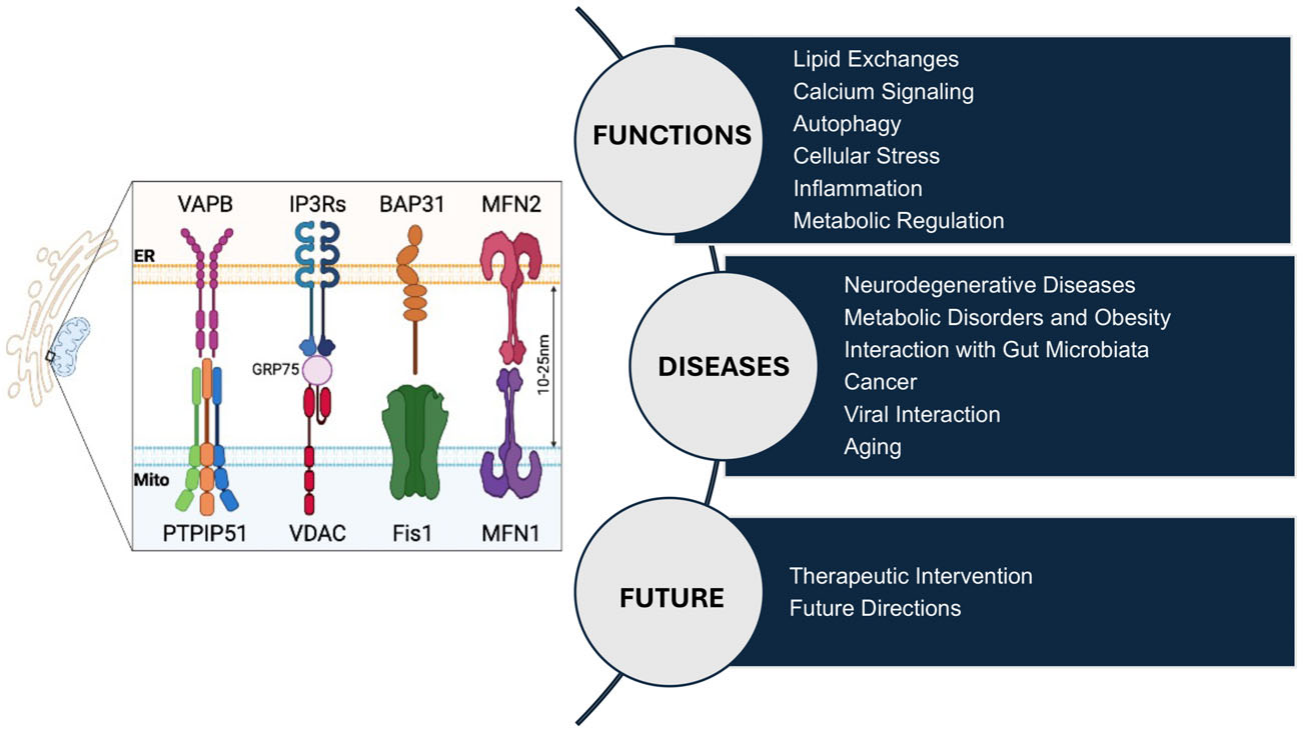
Mitochondria‐associated membrane (MAM) tethering proteins, functions, and impacts on disease. The figure provides an overview of the key tethering proteins that mediate MAM formation and their essential functions, including calcium signaling, mitochondrial metabolism, and lipid homeostasis. It also illustrates how MAM dysfunction contributes to the pathogenesis of several diseases, such as neurodegenerative disorders and aging. It also highlights potential therapeutic interventions aimed at restoring MAM integrity, offering future directions for research in targeting MAMs to mitigate disease progression.

MAMs are also regulated by specialized signaling proteins. The sigma‐1 receptor (Sig‐1R) functions as a calcium‐sensitive chaperone, stabilizing IP3R under stress conditions and modulating calcium signaling [7]. The NOD‐, LRR‐, and pyrin domain‐containing protein 3 (NLRP3) inflammasome is recruited to MAMs during activation, establishing these domains as central players in innate immunity and inflammation [8].

Functionally, MAMs serve as platforms for multiple processes:

1. Lipid synthesis and transfer—MAM‐localized enzymes synthesize phosphatidylserine (PS), which is transferred to mitochondria for conversion into phosphatidylethanolamine (PE) [9].
2. Calcium homeostasis—Calcium transfer through the IP3R–GRP75–VDAC1 complex regulates mitochondrial metabolism and apoptosis [10].
3. Mitochondrial dynamics—MAM‐associated proteins coordinate mitochondrial fission and fusion [11].
4. Autophagy—MAMs provide membranes and signaling platforms for autophagosome formation [12].
5. Stress responses—Including coordination of the unfolded protein response (UPR) and activation of inflammasomes.

Dysfunction in these MAM‐mediated processes contributes to a wide range of pathologies:

- Alzheimer's disease (AD)—altered MAM spacing promotes amyloid‐β (Aβ) production [13].
- Parkinson's disease (PD)—accumulation of alpha‐synuclein (α‐syn) at MAMs impairs PS metabolism [14].
- Metabolic diseases—MAM disruption affects insulin signaling and energy homeostasis [15].
- Viral infections—Viruses exploit MAM machinery to promote replication [16].

Given the essential role of MAMs in cellular homeostasis and their involvement in various diseases, understanding their molecular structure and regulatory mechanisms is critical. This review synthesizes current insights into MAM biology—from structural organization to functional roles—and highlights emerging therapeutic opportunities targeting MAM‐associated dysfunctions.

## Functions and cellular significance

### Lipid transfer and membrane homeostasis

Mitochondria‐associated membranes are specialized contact sites between the ER and mitochondria that support lipid synthesis and interorganelle transfer, crucial for maintaining membrane composition and function [2]. These structures maintain a narrow spacing (10–50 nm) that enables lipid exchange without fusion. Key tethering components form a crucial tripartite complex: the voltage‐dependent anion channel (VDAC1) on mitochondria is physically linked to the ER‐localized inositol 1,4,5‐trisphosphate receptor (IP3R) through the molecular chaperone GRP75, which serves as a bridge between these channels. Additional structural regulators—Mitofusin 2 (MFN2), VAPB, PTPIP51, and PACS2—maintain MAM stability and organization [9, 17].

MAMs are enriched in enzymes that synthesize phosphatidylserine (PS), which is transferred to mitochondria and converted to phosphatidylethanolamine (PE), contributing ~45% of mitochondrial PE. Mitochondria also return newly synthesized PE to the ER, where it serves as a precursor for phosphatidylcholine (PC) synthesis. Cardiolipin, synthesized exclusively in mitochondria, is trafficked to peroxisomes and lysosomes to influence membrane dynamics and function [18]. MAMs also mediate the exchange of PC, cholesterol, and sphingolipids, and regulate cardiolipin synthesis—a mitochondria‐specific phospholipid critical for energy production [9, 19, 20, 21, 22].

These lipid transfers regulate membrane fluidity, protein distribution, and respiratory complex organization. Cardiolipin enrichment, for instance, supports the assembly of respiratory supercomplexes and enhances oxidative phosphorylation [23, 24]. Disruption of MAMs reduces PE levels by up to 30%, mislocalizes cardiolipin, disrupts electron transport, impairs ATP production by up to 40%, and leads to mitochondrial fragmentation [25, 26].

MAMs also coordinate lipid droplet formation by linking ER lipid synthesis to storage pathways. Their disruption reduces lipid droplet formation by ~60%, exacerbating cellular stress and lipid dysregulation [24, 27].

Given their essential roles in lipid balance, mitochondrial function, and organelle communication, MAMs are emerging as therapeutic targets. Pharmacological modulation of MAM integrity may provide novel strategies for metabolic and neurodegenerative diseases. Understanding MAM‐regulated lipid dynamics is critical for leveraging this potential. While lipid transfer establishes MAM structure, calcium signaling across these contact sites is central to metabolic regulation and cell survival.

### Calcium signaling and metabolism

MAMs serve as key regulators of calcium signaling, supporting ATP production, apoptosis, and metabolic stability [28]. Calcium transfer from the ER to mitochondria is mediated by the same IP3R–GRP75–VDAC1 complex [29]. This coupling ensures efficient calcium flux while avoiding overload [6, 30].

Within mitochondria, calcium acts as a cofactor for enzymes in the tricarboxylic acid cycle and oxidative phosphorylation, directly modulating ATP output [31]. MAM‐controlled calcium flux allows cells to dynamically adjust their bioenergetics. Excessive calcium transfer can open the mitochondrial permeability transition pore (mPTP), initiating apoptosis under stress [32, 33, 34].

Therapeutically, stabilizing the IP3R–GRP75–VDAC1 complex preserves mitochondrial function and cell viability. For example, intranasal insulin maintains this complex in neurons, offering neuroprotective effects [35, 36]. Similarly, Lipin1 enhances cognitive function in diabetic encephalopathy by restoring calcium homeostasis through MAM integrity [37].

MAM calcium signaling dysregulation is linked to cancer, heart disease, and neurodegeneration [38]. Its dual role in metabolism and stress response presents both a pathological vulnerability and a therapeutic opportunity [39]. In addition to regulating energy metabolism, MAMs also coordinate quality control pathways like autophagy and mitophagy.

### Autophagy and mitochondrial quality control

MAMs coordinate autophagy and mitophagy—key quality control pathways that clear damaged organelles and proteins, supporting homeostasis [12].

Autophagy eliminates dysfunctional cellular components. MAMs promote this process under stress (e.g., oxidative stress or starvation) by regulating calcium release via the IP3R–GRP75–VDAC1 complex, which is essential for autophagosome biogenesis and maturation [40, 41].

MAMs also support mitophagy—the selective removal of damaged mitochondria. These sites serve as platforms for mitochondrial tagging and autophagosome formation [39, 42]. Their spatial organization ensures selective degradation of impaired mitochondria, preserving healthy ones and maintaining energy production.

In neurodegeneration, MAM fragmentation impairs autophagy. Kulkarni *et al*. [43] showed that loss of MAM scaffolding reduces autophagic flux in AD and PD. Pharmacological modulation of MAMs may restore autophagy; for instance, metformin enhances autophagic activity by modulating the IP3R–GRP75–VDAC1 tether, increasing cytosolic calcium in hepatocellular carcinoma [44, 45]. Although this was shown in cancer, the mechanism may apply broadly to autophagy‐impaired diseases.

Through their regulation of autophagy and mitophagy, MAMs help cells adapt to stress by removing dysfunctional components and supporting metabolic integrity. This quality control is vital under pathophysiological conditions [46, 47]. MAMs thus integrate stress signaling and degradation pathways to preserve cellular function. In parallel, MAMs serve as key responders to cellular stress, modulating both the UPR and integrated stress response (ISR) to restore homeostasis.

### Cellular stress responses

Mitochondria‐associated membranes are central to cellular stress response mechanisms, contributing to both the UPR and the ISR. These pathways are essential for cellular adaptation and survival under challenging conditions, and MAMs play a key role in coordinating these processes.

#### Unfolded protein response (UPR)

When the ER accumulates misfolded or unfolded proteins, the UPR is activated to restore ER homeostasis [48]. MAMs facilitate this response by serving as hubs for calcium and lipid exchange between the ER and mitochondria, regulated by proteins, such as MFN2 and PACS2 [49]. Calcium transfer at MAMs modulates three ER stress sensors—inositol‐requiring enzyme 1 (IRE1), protein kinase R‐like ER kinase (PERK), and activating transcription factor 6 (ATF6)—which initiate signaling pathways that enhance protein folding and reduce ER load [50]. MAM integrity directly influences both the activation and duration of these pathways, and disrupted contacts can prolong ER stress and trigger apoptosis [51].

#### Integrated stress response (ISR)

The ISR is triggered by diverse stressors including nutrient deprivation, viral infection, and ER dysfunction [52]. MAMs influence this pathway through their regulation of PERK, which also links UPR and ISR signaling. Upon activation, PERK phosphorylates eukaryotic initiation factor 2α (eIF2α), reducing global protein synthesis to conserve resources and promote recovery [53]. Calcium and lipid signaling at MAMs modulate PERK activity and thus impact the broader ISR.

Beyond protein folding and translation control, MAMs influence calcium homeostasis, mitochondrial energy metabolism, and lipid dynamics. Dysregulation of MAMs can compromise these processes and lead to cell death.

In summary, MAMs integrate and regulate UPR and ISR signaling, enabling cells to adapt to stress. Their ability to modulate overlapping yet distinct pathways highlights their role in maintaining cellular resilience.

### MAMs and inflammation

MAMs are critical regulators of inflammation, particularly through NLRP3 inflammasome activation and calcium‐dependent immune signaling [54, 55]. The NLRP3 inflammasome localizes to MAMs during activation [8], where these contact sites provide platforms for assembling the inflammasome complex and initiating inflammatory responses. Disrupted calcium handling at MAMs alters cytokine production and immune cell behavior [56].

#### Immune cell activation and NLRP3 regulation

In macrophages, MAM‐localized inflammasome activation requires both mitochondrial reactive oxygen species (ROS) and calcium signaling [57]. The spatial architecture of MAMs integrates these signals, facilitating the assembly of inflammasome components and the production of IL‐1β and IL‐18 [58]. Disruption of this coordination can drive excessive inflammation.

#### Reactive oxygen species (ROS)

MAM dysfunction increases mitochondrial ROS, a potent activator of the NLRP3 inflammasome. ROS also enhance NF‐κB activity and promote inflammasome oligomerization [13, 59], creating a self‐reinforcing inflammatory loop.

#### ER stress and inflammation

As discussed above, ER stress is modulated at MAMs. Unresolved ER stress can escalate inflammation or lead to cell death, linking MAM dysfunction with broader inflammatory signaling [49].

#### Mitochondrial DNA release

Dysfunctional MAMs may promote mitochondrial DNA (mtDNA) leakage into the cytoplasm, which activates innate immunity via the cGAS–STING pathway and contributes to chronic inflammation [60].

The interplay between MAM integrity and inflammation is increasingly relevant in neuroinflammatory, metabolic, and autoimmune diseases. Clarifying these mechanisms may open therapeutic strategies for controlling maladaptive inflammation.

### Metabolic regulation and disease

MAMs are key regulators of lipid metabolism and insulin signaling– both essential to energy homeostasis. They mediate phospholipid transfer between the ER and mitochondria, sustaining membrane composition and mitochondrial integrity [23]. Disruption of these exchanges contributes to ectopic lipid accumulation in the liver and muscle, driving metabolic disorders, such as nonalcoholic fatty liver disease (NAFLD) and insulin resistance [15, 61, 62].

MAMs also influence insulin signaling by hosting interactions between insulin receptor substrates and downstream signaling proteins [63]. They modulate calcium flux and impact the activity of Akt, a central insulin‐signaling kinase [64]. In conditions like chronic overnutrition, MAM expansion alters calcium signaling and impairs insulin action [65]. Loss of MAM integrity has been shown to reduce insulin sensitivity by up to 40% in skeletal muscle cells [66].

By orchestrating lipid dynamics, calcium homeostasis, and insulin responsiveness, MAMs stand at the intersection of cellular metabolism and disease. Restoring MAM function may offer a promising strategy for treating obesity, insulin resistance, and related metabolic disorders.

## Role in disease and therapeutic implications

### Neurodegenerative and neurocognitive disorders

Mitochondria‐associated ER membranes (MAMs) are increasingly recognized as critical regulators in neurodegenerative diseases, such as AD and PD, as well as neurocognitive and psychiatric disorders. The strategic positioning of MAMs at the interface between the ER and mitochondria makes them critical regulators of multiple cellular processes essential for neuronal survival and function.

#### Classical neurodegeneration (AD, PD)

Disrupted MAMs impair mitochondrial fission and fusion dynamics, contributing to neuronal degeneration [67, 68]. MAM dysfunction is increasingly recognized as a key factor that disrupts cellular homeostasis and leads to neuronal damage [69].

Recent evidence suggests that MAM integrity is compromised early in disease progression, potentially serving as an initiating event rather than merely a consequence of neurodegeneration [70].

Zellmer *et al*. [71] showed that narrow MAM contacts increase amyloid beta (Aβ) production and reduce mitochondrial motility in 3D AD neural cultures, while Barbuti *et al*. [72] found that α‐synuclein localizes to MAMs and disrupts phosphatidylserine metabolism, contributing to regional vulnerability in PD. Additionally, MAM disruption impairs mitophagy, as Luo *et al*. [73] demonstrated, exacerbating neurodegeneration in AD models.

**Fig. 2:**
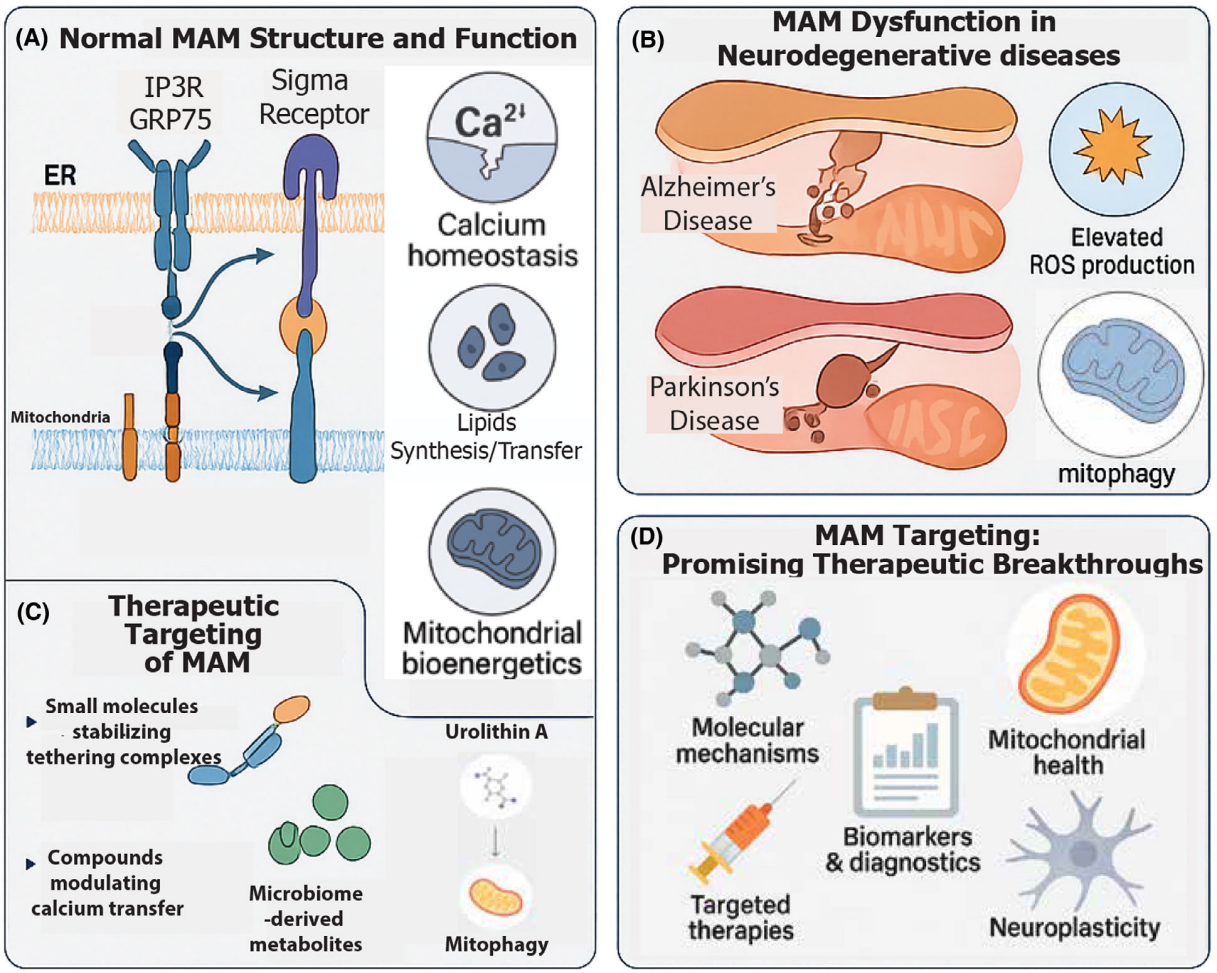
Mitochondria‐associated membrane (MAM) structure, dysfunction, and therapeutic targeting in neurodegenerative diseases. Comprehensive illustration of MAMs in health, disease, and therapeutic intervention. (A) Normal MAM structure showing key tethering complexes including IP3R–GRP75–VDAC1 and sigma‐1 receptor, which maintain proper calcium homeostasis and lipid transfer between the endoplasmic reticulum and mitochondria. (B) MAM dysfunction in neurodegenerative diseases, depicting disease‐specific alterations including narrowed contacts with increased Aβ production in Alzheimer's disease, α‐synuclein accumulation at MAMs in Parkinson's disease, elevated reactive oxygen species (ROS) production, and impaired mitophagy. (C) Therapeutic targeting of MAMs through multiple approaches including small molecules that stabilize tethering complexes, compounds that modulate calcium transfer, microbiome‐derived metabolites like urolithin A, and interventions enhancing mitophagy and mitochondrial bioenergetics. (D) Emerging therapeutic breakthroughs targeting MAMs, highlighting molecular mechanisms, mitochondrial targets, targeted delivery systems, biomarker development for early diagnosis, and approaches to enhance neuroplasticity through MAM modulation.

MAMs are central to calcium homeostasis; their dysfunction leads to excessive calcium transfer to mitochondria, resulting in calcium overload, oxidative stress, and neuronal damage [74, 75]. The impact of MAM dysfunction extends beyond mitochondrial dynamics to affect calcium homeostasis, lipid metabolism, and autophagy—all critical processes for maintaining neuronal health. Disruptions in these pathways create a self‐perpetuating cycle of cellular damage that accelerates disease progression [76]. Furthermore, MAM alterations appear to exhibit regional specificity within the brain, potentially explaining the selective vulnerability of certain neuronal populations in different neurodegenerative conditions [77] (Fig. 2).

#### Neurocognitive and psychiatric disorders

MAM dysfunction extends beyond classical neurodegenerative diseases to neurocognitive and psychiatric disorders, revealing common pathophysiological mechanisms across neurological conditions. MAMs regulate calcium dynamics in neurons, influencing key processes, such as synaptic plasticity, neurotransmitter release, and neuronal excitability, all of which are essential for learning and memory [77].

Zhang *et al*. [78] demonstrated in mouse models of depression that chronic stress enhances MAM contact formation and ER–mitochondria calcium transfer in microglia, leading to NLRP3 inflammasome activation and depressive behaviors. This study implicates MAMs in microglial stress responses, further linking MAM dysfunction to neurocognitive deficits.

In schizophrenia, MAM dysfunction contributes to aberrant calcium signaling, mitochondrial impairment, and oxidative stress that underlie the cognitive deficits characteristic of the disorder [79]. Recent investigations have revealed that genetic risk factors for schizophrenia, including DISC1 and NRG1, interact with MAM components, potentially explaining the mitochondrial abnormalities observed in patient‐derived neurons [80].

Alterations in MAM‐mediated mitochondrial function and calcium signaling have been strongly associated with mood disorders, including depression and bipolar disorder [81]. Zhang *et al*. [78] highlighted that disrupting GRP75, a key protein in MAM formation, reversed stress‐induced MAM changes and depressive behaviors. Yang *et al*. [82] discussed the potential of natural products targeting MAMs as antidepressant therapies, while studies show that antidepressants can normalize MAM‐associated calcium signaling in preclinical models [83].

#### Therapeutic approaches and future directions

The recognition of MAM involvement across neurodegenerative and neurocognitive disorders has opened new therapeutic avenues targeting these specialized membrane contacts. Advancements in precision medicine may enable personalized treatments by targeting MAM‐related pathways, improving therapeutic efficacy while minimizing adverse effects. The identification of patient‐specific MAM alterations through advanced imaging and molecular profiling could guide tailored therapeutic strategies.

Research has explored targeting MAM‐associated pathways to restore calcium signaling and mitochondrial function [84]. In ischemic stroke models, downregulation of the VAPB‐PTPIP51 tether reduced MAM integrity, but activation of the PI3K pathway reversed these effects [85]. Etxebeste‐Mitxeltorena *et al*. [86] identified small molecules that enhance MAM contact, restoring lipid metabolism in ALS patient cells and improving mitochondrial morphology.

Emerging technologies such as CRISPR‐based approaches offer unprecedented opportunities to correct specific genetic defects affecting MAM function [87]. Small molecule modulators of MAM integrity and function are being developed as potential disease‐modifying agents that could be selected based on individual patient profiles. Advanced imaging technologies, including super‐resolution microscopy and cryo‐electron tomography, are deepening our understanding of MAM structure and function with unprecedented detail [88]. Zellmer *et al*. [89] developed quantitative tools using live‐cell imaging to measure MAM dynamics in 3D neural models, providing methods to assess MAM‐targeting therapeutics.

Biomarker development focused on MAM dysfunction may facilitate early intervention before irreversible neuronal damage occurs. Circulating markers of MAM stress could potentially be detected in blood or cerebrospinal fluid, enabling noninvasive monitoring of disease progression and treatment response [90]. Multi‐omics approaches integrating proteomics, lipidomics, and metabolomics may identify MAM‐associated signatures that predict disease onset and progression.

### Metabolic disorders and obesity

MAMs are essential regulators of cellular metabolism, particularly in lipid homeostasis. Their disruption contributes to metabolic syndromes, such as obesity, insulin resistance, and NAFLD, largely through impaired mitophagy, defective mitochondrial quality control, and increased oxidative stress [15, 91].

Therapeutic strategies that target MAMs to enhance calcium signaling and lipid exchange are emerging. Preclinical studies indicate that restoring MAM function can improve metabolic balance and insulin sensitivity in obesity and diabetes models [61, 92]. Drug discovery efforts focused on MAM—associated proteins and lipids are yielding promising candidates for clinical development, including compounds that stabilize MAM contacts, enhance calcium buffering capacity, or improve mitochondrial quality control [93].

### Interaction with gut microbiota

**Fig. 3:**
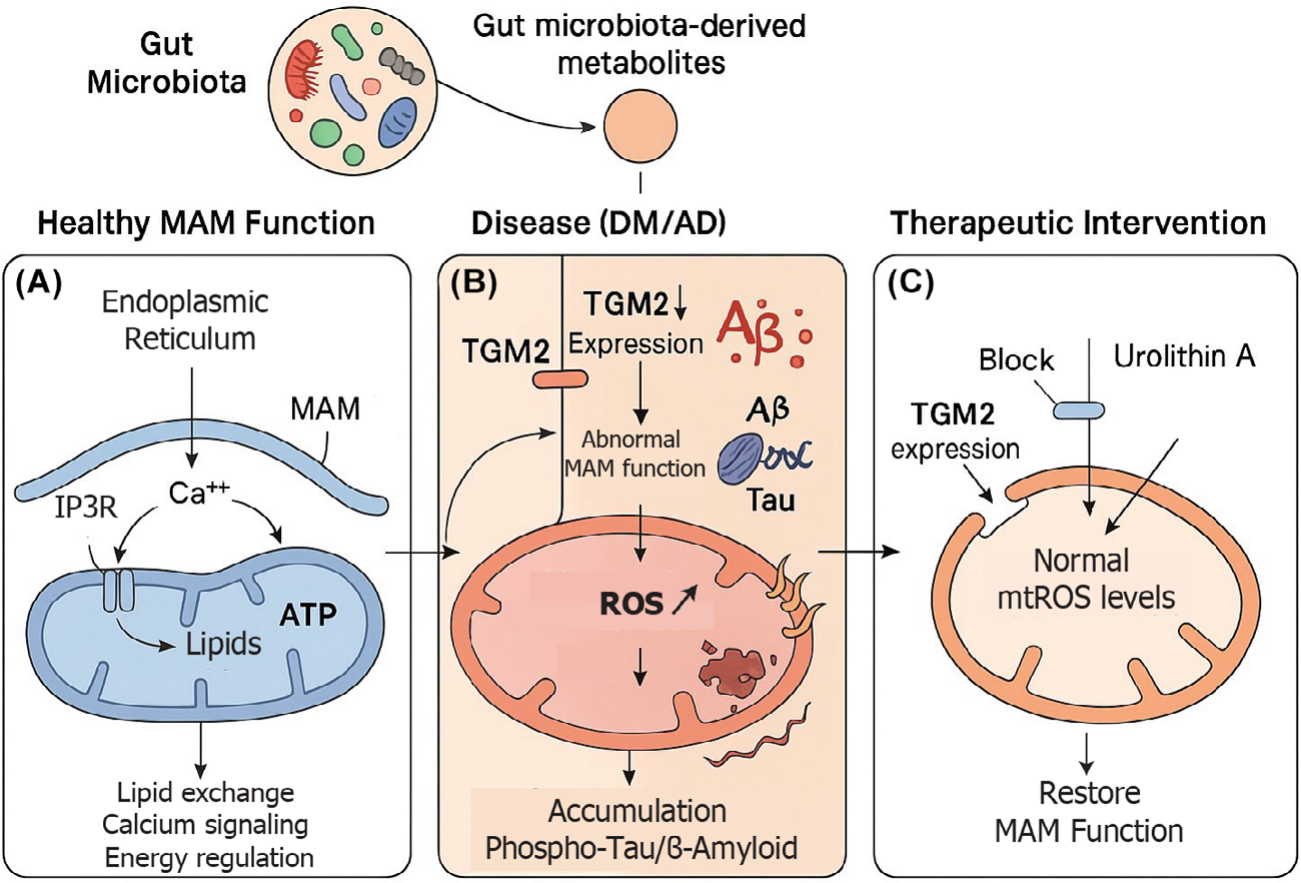
Gut microbiota‐derived metabolites modulate mitochondria‐associated membrane (MAM) function in neurodegenerative and metabolic disorders. Schematic illustration of the interplay between gut microbiota, MAMs, and neurodegeneration. (A) In healthy conditions, gut microbiota‐derived metabolites support normal MAM function, facilitating proper calcium signaling, lipid exchange, and energy regulation between the endoplasmic reticulum (ER) and mitochondria via the IP3R–GRP75–VDAC1 complex. (B) In disease states such as diabetes mellitus (DM) and Alzheimer's disease (AD), increased transglutaminase type 2 (TGM2) expression disrupts MAM integrity, leading to elevated mitochondrial reactive oxygen species (ROS) production, abnormal calcium handling, and accumulation of pathological proteins (phospho‐tau and β‐amyloid). (C) Therapeutic intervention with urolithin A, a gut microbiota‐derived metabolite from ellagitannins, blocks TGM2 expression, normalizes mitochondrial ROS levels, and restores MAM function, potentially mitigating neurodegenerative processes. This mechanism represents a novel gut–brain axis pathway with implications for both metabolic and neurodegenerative disorders.

Recent research has uncovered links between gut microbiota and MAM function, providing a new dimension to host metabolic regulation [94]. Microbial metabolites, notably urolithin A produced from ellagitannins in the gut, modulate MAM‐related processes, such as mitochondrial calcium influx and ROS generation. Urolithin A reduces transglutaminase type 2 (TGM2) expression and MAM formation, thereby attenuating mtROS accumulation, suppressing Aβ‐producing enzymes, and improving cognitive outcomes in diabetic models (Fig. 3).

Investigating the interplay between MAMs and the gut microbiota may reveal new insights into disease pathology [94]. The discovery that microbial metabolites can influence MAM function opens exciting possibilities for dietary and probiotic interventions to modulate neurodegeneration and metabolic dysfunction.

### Cancer

MAMs contribute to cancer pathogenesis by facilitating metabolic reprogramming to support uncontrolled cell proliferation [95, 96]. They enhance energy production and biosynthesis, while also modulating calcium signaling and mitochondrial membrane potential to promote apoptosis resistance [97].

Targeting MAM‐associated pathways is emerging as a novel cancer treatment approach. Efforts include disrupting MAM‐mediated metabolic advantages and improving drug sensitivity [98, 99, 100, 101]. Advances in imaging technologies now allow high‐resolution visualization of MAMs, facilitating deeper insight into their roles in tumor biology [102, 103].

### Viral interactions and emerging threats

Since 2010, MAMs have been identified as key sites exploited by viruses to facilitate replication and immune evasion. Viruses such as hepatitis C and dengue manipulate MAM‐associated lipid and calcium transfer for replication [104, 105]. HIV‐1 hijacks MAMs at multiple stages of its life cycle, altering calcium and lipid dynamics to enhance viral assembly and budding [16, 106].

The COVID‐19 pandemic and other emerging pathogens like Zika virus have renewed interest in how viruses exploit MAMs [107, 108]. The sigma‐1 receptor (Sig‐1R), a chaperone protein enriched at MAMs, has emerged as a critical factor in viral infections, including SARS‐CoV‐2, making it an attractive target for broad‐spectrum antiviral development.

### Aging and senescence

Aging is a major risk factor for neurodegeneration, and age‐related MAM dysfunction contributes to impaired mitochondrial efficiency, calcium dysregulation, and oxidative stress [109, 110, 111, 112]. These deficits promote cellular senescence and inflammation, hallmarks of aging. Wang *et al*. [113] reported MAM alterations during aging, including disrupted ER–mitochondrial morphology and signaling.

MAMs regulate senescence‐associated mitochondrial function, with proteins like VAPB‐PTPIP51 playing key roles in aging‐linked decline [114, 115]. Targeting MAMs could slow age‐related cellular decline and improve resilience, representing a potential antiaging therapeutic strategy.

## Conclusion and future directions

MAMs serve as central orchestrators of cellular homeostasis, linking mitochondrial function, calcium signaling, and lipid metabolism to diverse pathological processes across neurodegenerative, metabolic, infectious, and neoplastic diseases. The emerging understanding of MAM dysfunction reveals common pathophysiological mechanisms that span seemingly distinct conditions, from AD to metabolic disorders to viral infections.

As research continues to elucidate the molecular details of MAM regulation, novel therapeutic strategies targeting MAM integrity and signaling hold promise for addressing complex disease challenges. The convergence of advanced imaging technologies with targeted pharmacological approaches may soon enable precision medicine approaches that restore MAM function in patient‐specific contexts. By targeting the fundamental cellular processes regulated by MAMs, future therapeutic strategies may achieve true disease modification rather than merely symptomatic relief, potentially revolutionizing treatment paradigms across multiple conditions and moving us closer to transformative advances in diagnosing, treating, and ultimately preventing these devastating diseases.

Future research on MAMs will further elucidate their role in cellular communication, disease mechanisms, and therapeutic strategies. Key areas of focus included the following:

1. Molecular mechanisms: Advanced imaging technologies, including super‐resolution microscopy and cryo‐electron tomography, will deepen our understanding of MAM structure and function in health and disease. These approaches will reveal the dynamic nature of MAM contacts and their regulation with unprecedented detail [88].
2. Biomarkers and diagnostics: Continued efforts in biomarker discovery will facilitate early disease detection and treatment monitoring [116]. Multi‐omics approaches integrating proteomics, lipidomics, and metabolomics may identify MAM‐associated signatures that predict disease onset and progression.
3. Targeted therapies: Drug discovery efforts focused on MAM‐associated proteins and lipids are yielding promising candidates for clinical development [93]. These include compounds that stabilize MAM contacts, enhance calcium buffering capacity, or improve mitochondrial quality control.
4. Gut–brain axis: The discovery that microbial metabolites can influence MAM function opens exciting possibilities for dietary and probiotic interventions to modulate neurodegeneration and metabolic dysfunction [94].
5. Neurocognitive function: Understanding how MAMs contribute to synaptic plasticity and neuronal excitability could lead to novel cognitive enhancement strategies and deeper insights into learning and memory mechanisms [79].

As research progresses, MAMs are poised to bridge critical gaps in our understanding, offering new perspectives on disease prevention and treatment. The convergence of advanced technologies will accelerate discoveries in this rapidly evolving field, potentially achieving true disease modification rather than merely symptomatic relief.

## Conflict of interest

The authors declare no conflict of interest.

## Authors contributions

VB, MS, NS, and BES contributed to manuscript editing. All authors approved the submitted version.

## Declaration of generative AI and AI‐assisted technologies

During the preparation of this work, the author(s) used ChatGPT in order to improve clarity, flow, and readability. AI was used exclusively for text editing and refinement, with no involvement in data analysis. After using this tool/service, the author(s) reviewed and edited the content as needed and take(s) full responsibility for the content of the publication.
